# Favipiravir for the treatment of COVID-19 in elderly patients—what do we know after 2 years of COVID-19?

**DOI:** 10.1007/s11357-022-00582-8

**Published:** 2022-05-11

**Authors:** Henrietta Papp, Zsófia Lanszki, György M. Keserű, Ferenc Jakab

**Affiliations:** 1 National Laboratory of Virology, University of Pécs, Pecs, Hungary; 2 Institute of Biology, Faculty of Sciences, University of Pécs, Pecs, Hungary; 3 Medicinal Chemistry, Research Center for Natural Sciences, Budapest, Hungary

**Keywords:** T-705, Geriatrics, Severe acute respiratory syndrome coronavirus 2, Clinical trials

## Abstract

Since the appearance of coronavirus disease 2019 (COVID-19), numerous studies have been conducted to find effective therapeutics. Favipiravir (FVP) is one of the repurposed drugs which has been authorized in a few countries on an emergency basis to treat COVID-19. Elderly individuals especially 65 years or older are more prone to develop severe illness. We aim to provide a short summary of the current knowledge of the antiviral efficacy of favipiravir with respect to severe acute respiratory syndrome coronavirus 2 (SARS-CoV-2)–infected elderly patients. We found that it is rather controversial whether favipiravir is effective against SARS-CoV-2 infection. Data regarding patients 65 years or older is not sufficient to support or reject the usage of favipiravir for COVD-19 treatment. Further studies would be advisable to elicit the efficiency of favipiravir in elderly COVID-19 patients.

## Introduction

Favipiravir (Avigan™, T-705, 6-fluoro-3-hydroxy-2-pyrazinecarboxamide) was approved in Japan in 2014 against emerging influenza viruses. However, it is a last-resort medication to treat the novel or re-emerging influenza viruses that are resistant to certain antivirals, like oseltamivir [1]. Its anti-influenza virus activity was discovered during an extensive research led by Toyama Chemical Co., Ltd. [2]. Since its discovery, numerous studies have been published about its effectiveness against different viruses. Besides different influenza virus strains (types A, B, and C), it potently inhibited the replication of various flavi-, noro-, alpha-, filo-, arena-, hanta-, and bunyaviruses both in vitro and in vivo [1, 3]. After cellular uptake, it is phosphoribosylated and recognized as a substrate for the viral RNA-dependent RNA polymerase (RdRp) and can cause chain termination or lethal mutagenesis [1, 4]. One major difference, compared to several other ribonuclease analogues, is the lack of mitochondrial toxicity, which is a known side effect of others such as R1479, INX-08189, NITD-008, and ribavirin in combination with didanosine [5, 6]. It was also demonstrated that favipiravir does not interfere with the activity of the DNA-dependent RNA polymerase (DdRp) [7]. In the case of antiviral drugs, one of the most significant questions is the emergence of resistance. Drug-resistant viruses from favipiravir-treated patients have not been identified so far. However, there are three studies where favipiravir-resistant mutants were obtained in vitro. All three resistant mutants carried mutations in their polymerase gene. In the case of influenza A and chikungunya virus, lysine to arginine and the enterovirus 71 serine to asparagine mutations were identified [8, 9]. Considering its broad-spectrum anti-RNA viral activity and low cytotoxicity, it is a promising agent against the newly emerged positive-sense RNA virus, SARS-CoV-2. Wang et al. investigated the in vitro antiviral activity of favipiravir against SARS-CoV-2 in the Vero E6 cell line. The half-maximal effective concentration (EC_50_) value of favipiravir was 61.88 µM (9.72 µg/ml) [10]. EC_50_ is routinely used to determine the potency of a compound. The EC_50_ concentration determines the 50% of maximal response. Presumably, favipiravir is inserted into the newly synthesized SARS-CoV-2 RNA chain, which caused a detrimental effect on the viral replication. Shannon et al. demonstrated a high occurrence of G-to-A and C-to-U transition mutations in the SARS-CoV-2 genome in the presence of 500 µM favipiravir [11]. These mutations following favipiravir treatment have been observed in other viruses as well [1]. In vivo studies are proving the anti-SARS-CoV-2 activity of favipiravir in a Syrian hamster model (*Mesocricetus auratus*) at 600–1400 mg/kg [12, 13]. Numerous investigative clinical trials and bioequivalence studies are ongoing and a few have been completed, and the results were published. Favipiravir has been approved at a fast pace for the treatment of mild to moderate COVID-19 for instance in China, Russia, India, Hungary, and Thailand. Studies that enrolled ≥ 65-year-old patients who had symptomatic SARS-CoV-2 infection and were treated with favipiravir are discussed here.

## Results

Favipiravir proved to be promising in several studies. Among the first reported results, Cai et al. compared favipiravir and lopinavir (LPV)/ritonavir (RTV) (ChiCTR2000029600) in 2020. Few patients were involved in this study (35 patients in the favipiravir arm, while 45 in LPV/RTV), and all received interferon α-1b besides the drugs (60 µg). According to the guidelines of the Chinese National Health Commission, all of the patients had moderate infections. Patients in the favipiravir arm received 1600 mg twice a day (BID) on day 1 and 600 mg BID from day 2 to day 14. The primary outcome was the viral clearance that was monitored by SARS-CoV-2-specific quantitative reverse transcription-polymerase chain reaction (qRT-PCR). Besides qRT-PCR, chest computed tomography (CT) was made to evaluate the efficacy of the drugs. The median time of viral clearance was 4 days and 11 days in the case of favipiravir- and LPV/RTV-treated patients, respectively. The results are not separated by age, so the effect and adverse events are not known exactly in elderly patients [14]. Ivashcenko et al. in 2020 conducted an open-label, randomized, adaptive study with 60 patients (NCT04434248). A percentage of 46.7% of the patients enrolled in this study were 60 or older and/or had concurrent chronic conditions. Patients received 1800 or 1600 mg BID favipiravir on the first day, then from the second day 800 or 600 mg BID. Patients in the control arm received standard of care (SOC) based on the guidelines of the Russian Federation for the treatment of COVID-19. The viral clearance rate was similar in both dosing regimens. Percentages of 62.5% and 92.5% of the favipiravir-treated patients had negative PCR results on day 5 and day 10, respectively, while on the fifth day 30% and on the tenth day 80% of the patients in the SOC arm achieved viral clearance. The body temperature of the favipiravir-treated patients normalized (< 37 °C) in 2 days (median time), and by 15 days, chest CT scans improved in 90% of the patients, while in the SOC arm body temperature normalization was achieved in 4 days (median time) and CT scan improvement on the fifteenth day was seen in 80% of the patients. The published report does not have data on how effective favipiravir was in ≥ 65-year-old patients. It would be beneficial for understanding the potential of favipiravir treatment for COVID-19 in geriatric patients [15]. The Japanese Association of Infectious Diseases also reported observations of favipiravir treatment in COVID-19 cases. In 2020, a 64-year-old patient with diabetes and hypertension received 1800 mg twice on the first day then 800 mg twice for 6 days. Fever has been relieved on the first day of the treatment, and then the oxygenation and dietary intake improved as well [16]. Alamer et al. conducted a retrospective study in 2020 and published their data in 2021. Their analyses showed that the median time to discharge was 10 days in the favipiravir-treated groups versus 15 days in the case of those patients who received supportive care, although in the case of ≥ 65-year-old patients there was no significant difference in the discharge events and mortality outcomes between the favipiravir-treated and SOC-treated patients [17]. Shinkai et al. also observed clinical improvement in moderate COVID-19 patients faster in the favipiravir-treated group (JapicCTI-205238). Patients received on day 1 1800 mg BID and from days 2 to 13 800 mg BID favipiravir or placebo. The ratio of the ≥ 65-year-old patients was higher in the placebo group, but the proportion of high-/medium-risk patients was higher in the favipiravir arm [18].

There are studies where favipiravir did not show significant improvement in the clinical recovery rate. Chen and his coworkers compared Arbidol (umifenovir) and favipiravir in a randomized, controlled, open-label trial (ChiCTR2000030254) in 2020. Umifenovir is also a viral RdRp inhibitor, which is used to treat influenza-associated pneumonia. Patients received standard care and Arbidol (200 mg 3 times a day) or favipiravir (on the first day 1600 mg BID then 600 mg BID) for 10 days. The authors defined the clinical recovery as the continuous recovery of pyrexia, respiratory rate ≤ 24 times/min, oxygen saturation ≥ 98%, and cough relief. They did not find a significant difference in the clinical recovery rate at day 7. However, favipiravir significantly shortened the fever and coughing period in the case of moderate infections [15]. Fujita Health University released an observational study in 2020, in which they reported that among the elderly patients, improvement was documented in fewer cases than in younger adults; moreover, worsening of the symptoms was observed more frequently in the 60-year-older patients. A total of 2158 patients were treated with favipiravir. The majority of the patients were older than 50 years old in this study. The majority of the subjects got 1800 mg twice on the first day then 800 mg twice on the consecutive days. Of the favipiravir-treated patients, 41.6% received ciclesonide and 3.4% took lopinavir/ritonavir; 52.3% were older than 60 years. The median length of the therapy was 11 days [19]. According to a retrospective, observational study in Thailand, favipiravir is effective against COVID-19, although older age proved to be a poor prognostic factor for day 7 clinical improvement [20]. In 2021, Szabo et al. published a report where the median age of the enrolled patients was 66.0 ± 12.4 years; moreover, in the favipiravir arm, the median age was 71.5 ± 15.1 years. Favipiravir treatment did not show a statistically significant difference in disease progression between the cohorts of moderate-to-severe COVID-19 patients. Moreover, the patients in the favipiravir cohort needed immunomodulatory therapy more often than the patients in the non-favipiravir cohort [21].

Adverse effects (AE) were observed during favipiravir treatment. The most common was the elevated serum uric acid (SUA) level (> 6 mg/dL for women, > 7 mg/dL for men, and > 5.5 mg/dL for under 18 years old children) [17, 18, 22–26], although symptoms did not manifest in the reported cases, and the SUA level turned back to normal after discontinuing the favipiravir therapy. But caution has to be taken as increased SUA level can pose risk to patients who have a history of gout, dissatisfactory kidney function, and hyperuricemia and can also be a risk factor in patients at high cardiovascular risk [25, 27–30]. Prolongation of the heart rate–corrected QT (QTc) interval (≥ 0.45 s for men and ≥ 0.47 s for women) has been observed during exceptionally high-dose favipiravir treatment in the case of an Ebola-infected patient [31]. However, the Ebola-infected patient received 6 g favipiravir on the first day and 1.2 g twice daily for 9 days. It is a higher dose compared to a Japanese study where patients received a single oral dose of favipiravir 1.2 g or 2.4 g. In this study, a prolonged QT/QTc interval was not observed [32]. However, in this study, the subjects were young (21–38 years old), healthy adults. Besides, only a single dose of favipiravir was administered, while the therapy of COVID-19 patients took a longer period of time (10–14 days).

## Discussion

Trials that examined favipiravir as a treatment for COVID-19 generally had a low sample size; moreover, the proportion of the ≥ 65-year-old patients involved in the trials was very low. Furthermore, the studies differ for instance in the length of the treatment, dosage, proportions of different age groups, and co-morbidities. Also, the seven-point ordinal scale was rarely utilized to assess the efficacy. Therefore, it is rather difficult to fully understand the effectiveness of favipiravir. Meta-analyses might be a good tool to unravel the potential of favipiravir for the COVID-19 treatment from the data already obtained from the different trials. Manabe et al. conducted a meta-analysis with studies that were published by the end of 2020. The analysis revealed that in the case of mild- to moderate COVID-19 patients favipiravir treatment significantly assists the viral clearance by the seventh day of the treatment, although on the fourteenth day the difference is not significant. Favipiravir-treated patients also exhibited a significantly better clinical improvement both by the seventh and fourteenth days [33]. Hassanipour et al. published a systematic review and meta-analysis of clinical trials in August 2021; it includes 9 studies. They found significant clinical improvement in the case of favipiravir-treated patients after 7 and 14 days of hospitalization, like Manabe et al. But they did not find a statistical difference between the favipiravir-treated and control groups on viral clearance, transfer to intensive care unit, supplementary oxygen requirement, adverse events, and mortality rate [26]. In addition, Özlüşen et al. also performed a meta-analysis. Their study focuses on eliciting the favipiravir efficacy for the treatment of moderate to severe COVID-19. They did not find a significant difference between the patients receiving favipiravir or SOC on fatality rate and the requirement of mechanical ventilation [34].

In addition to vaccines, antiviral treatment is also needed to mitigate the effects of the coronavirus epidemic. This is justified by the high number of unvaccinated people, the possible infection of the vaccinated population and the infections they induce, the time limit of vaccinees’ immunity, and the emergence of new virus variants. Antiviral treatment is of particular importance in groups of patients affected by COVID-19 and associated diseases (diabetes, chronic heart failure, cerebrovascular accident, chronic lung disease, etc.), who are therefore at increased risk. Elderly patients are among the most vulnerable and most frequently hospitalized patients [35, 36].

The place of effective antiviral therapy is in the viral phase of the disease, 7–10 days after infection, within 5 days of onset of symptoms. At this time, the majority of patients are at home (outpatients) and only appropriate oral antiviral therapy can be considered. In the first 2 years of the COVID pandemic, favipiravir was the only available home treatment. Favipiravir is a broad-spectrum antiviral compound that effectively inhibits the replication of the SARS-CoV-2 virus in vitro and in animals in vivo. According to several validated reports, early favipiravir treatment reduces viral load in infected patients, thus accelerating recovery. However, studies published at the end of 2021 showed that favipiravir treatment was not sufficiently effective in studies with larger numbers of patients. Literature data suggest that the antiviral efficacy of favipiravir is limited, depending on when and for how long treatment is started and the dose used. New options for antiviral treatment for outpatients are molnupiravir and Paxlovid, which, according to published test results, are more effective at lower doses than favipiravir [37, 38]. Further investigations regarding the effectiveness of different therapeutics in the elderly population should be of high priority for future research.
